# CRISPR/Cas9 assisted stem cell therapy in Parkinson's disease

**DOI:** 10.1186/s40824-023-00381-y

**Published:** 2023-05-16

**Authors:** Poojitha Pinjala, Kamatham Pushpa Tryphena, Renuka Prasad, Dharmendra Kumar Khatri, Woong Sun, Shashi Bala Singh, Dalapathi Gugulothu, Saurabh Srivastava, Lalitkumar Vora

**Affiliations:** 1 Molecular and Cellular Neuroscience Lab, Department of Pharmacology and Toxicology, National Institute of Pharmaceutical Education and Research (NIPER)-Hyderabad, Telangana-500037, Hyderabad, India; 2 Department of Anatomy, Korea University College of Medicine, Moonsuk Medical Research Building, 73 Inchon-Ro, Seongbuk-Gu, Seoul, 12841 Republic of Korea; 3 Department of Pharmaceutics, Delhi Pharmaceutical Sciences and Research University (DPSRU), New Delhi, 110017 India; 4 Department of Pharmaceutics, National Institute of Pharmaceutical Education and Research (NIPER)-Hyderabad, Telangana-500037, Hyderabad, India; 5 School of Pharmacy, Queen’s University Belfast, 97 Lisburn Road, Belfast, BT9 7BL UK

**Keywords:** Neurodegenerative disorder, α-synuclein, Gene editing, Human pluripotent stem cells, Embryonic stem cells, Disease model

## Abstract

Since its discovery in 2012, CRISPR Cas9 has been tried as a direct treatment approach to correct the causative gene mutation and establish animal models in neurodegenerative disorders. Since no strategy developed until now could completely cure Parkinson's disease (PD), neuroscientists aspire to use gene editing technology, especially CRISPR/Cas9, to induce a permanent correction in genetic PD patients expressing mutated genes. Over the years, our understanding of stem cell biology has improved. Scientists have developed personalized cell therapy using CRISPR/Cas9 to edit embryonic and patient-derived stem cells *ex-vivo*. This review details the importance of CRISPR/Cas9-based stem cell therapy in Parkinson's disease in developing PD disease models and developing therapeutic strategies after elucidating the possible pathophysiological mechanisms.

**Graphical Abstract:**

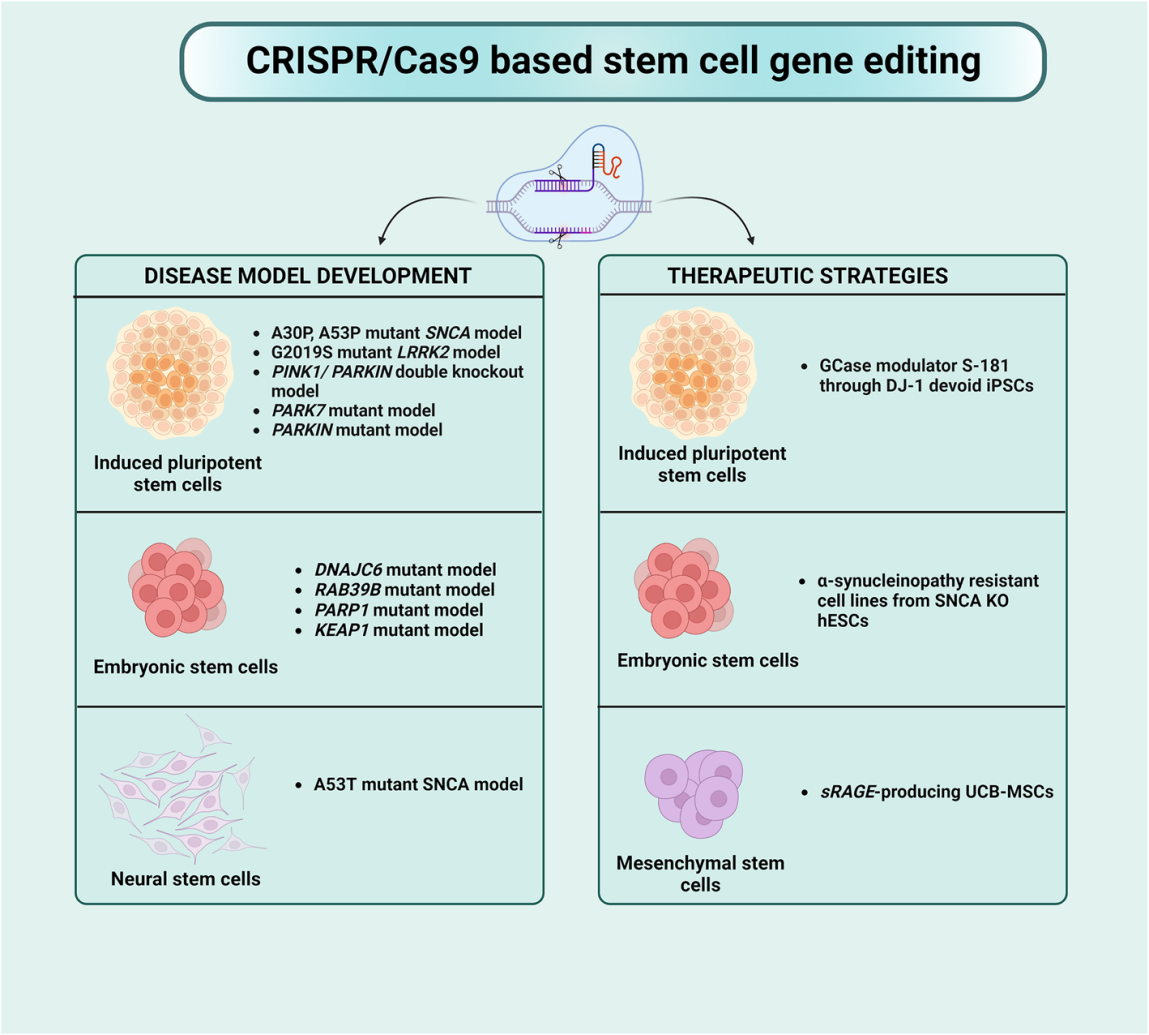

## Background

Parkinson's disease (PD) is a complex and multifocal neurodegenerative disorder with pathogenesis originating from the synergy of abnormal α-synuclein aggregation, neuroinflammation, and dysfunction of mitochondria, lysosomes, and synaptic transport issues influenced by genetic and idiopathic factors. Monogenic forms of PD are characterized by Lewy body development due to cellular dysfunction and degeneration of the dopaminergic neurons in the substantia nigra pars compacta (SNpc) [1]. It starts by affecting reasoning, circadian rhythms, and autonomous functioning, followed by Parkinsonian motor symptoms such as muscle rigidity, hypokinesia, tremors, and impaired postural stability, which worsen with time [2].

Based on whether the treatment targets the underlying cause of the disease or relieves its symptoms, they can be categorized into disease-modifying and non-disease-modifying therapies. Non-disease modifying therapy aims to revert dopaminergic functioning to not only restrict symptoms but also decelerate the progression of the disease by either boosting dopamine levels or activating dopamine receptors in the brain. However, with prolonged use, adverse effects such as dyskinesia, insomnia, and hallucinations eclipse the positive impact of these drugs. Due to these drawbacks, extensive research is being done to identify the disease-modifying therapies that could approach the exact target of the disease. New pharmacological targets are being identified, and by applying the concept of gene therapy targets, stem cell transplantation, and surgical interventions, the delivery of drugs to affected neurons can be achieved [3].

Among these, gene editing is the most appropriate substitute for traditional disease-modifying therapy. While it is currently not possible for neurodegenerative disorders to be treated, gene editing has the potential to do so. Over the years, with advances in technology, the development of gene editing technologies such as zinc-finger nucleases (ZFNs) and transcription activator-like effector nucleases (TALENs) has materialized [4, 5], in which various nucleases can identify and cleave intended DNA sequences. Although they indicated the beginning of precise gene editing, when applied therapeutically, they turned out to be expensive because of the complex design of the nuclease [6]. The discovery of the clustered, regularly interspaced short palindromic repeats (CRISPR/Cas9) system is a significant scientific revolution. CRISPR/Cas9 is the most prominent and powerful tool due to its higher specificity and efficiency [4, 5]. Thus, CRISPR/Cas9-based gene therapy has reached the clinical trial stage for many monogenic diseases such as sickle cell anemia [7], β-thalassemia [7], hereditary tyrosinemia type I [8] and is being applied in advanced preclinical testing stages of Duchenne muscular dystrophy (DMD), hemoglobinopathies, and hereditary tyrosinemia type I. Gene therapy has been beneficial in neurodegenerative diseases such as Alzheimer's disease [9-11] and Parkinson's disease [12-14] as well.

Due to stem cells' anti-inflammatory and anti-apoptotic properties, stem cell therapy is a promising approach to treat neurodegenerative diseases, especially PD, whose pathogenesis is linked to factors such as inflammation and neuronal differentiation [15]. The aim is to restore or replace dysfunctional dopaminergic neurons and rescue abnormal motor functions. Hence, by merging the benefits of stem cells and the CRISPR/Cas9 tool, studies are being designed to create disease models, perform gene correction, generate PD-resistant cells, and execute screening to identify potential therapeutic compounds. A combination of stem cells and CRISPR/Cas9 improves the functionality of homology-directed repair (HDR) machinery through increased activation, which can help avoid unnecessary off-target mutations [16]. Although ethical concerns have been frequently raised, stem cells are being utilized in different studies because their characteristics, such as unlimited self-renewal and the ability to differentiate into specialized adult cell types, have outweighed their disadvantages [17].

## An overview of Parkinson's disease

**Fig. 1:**
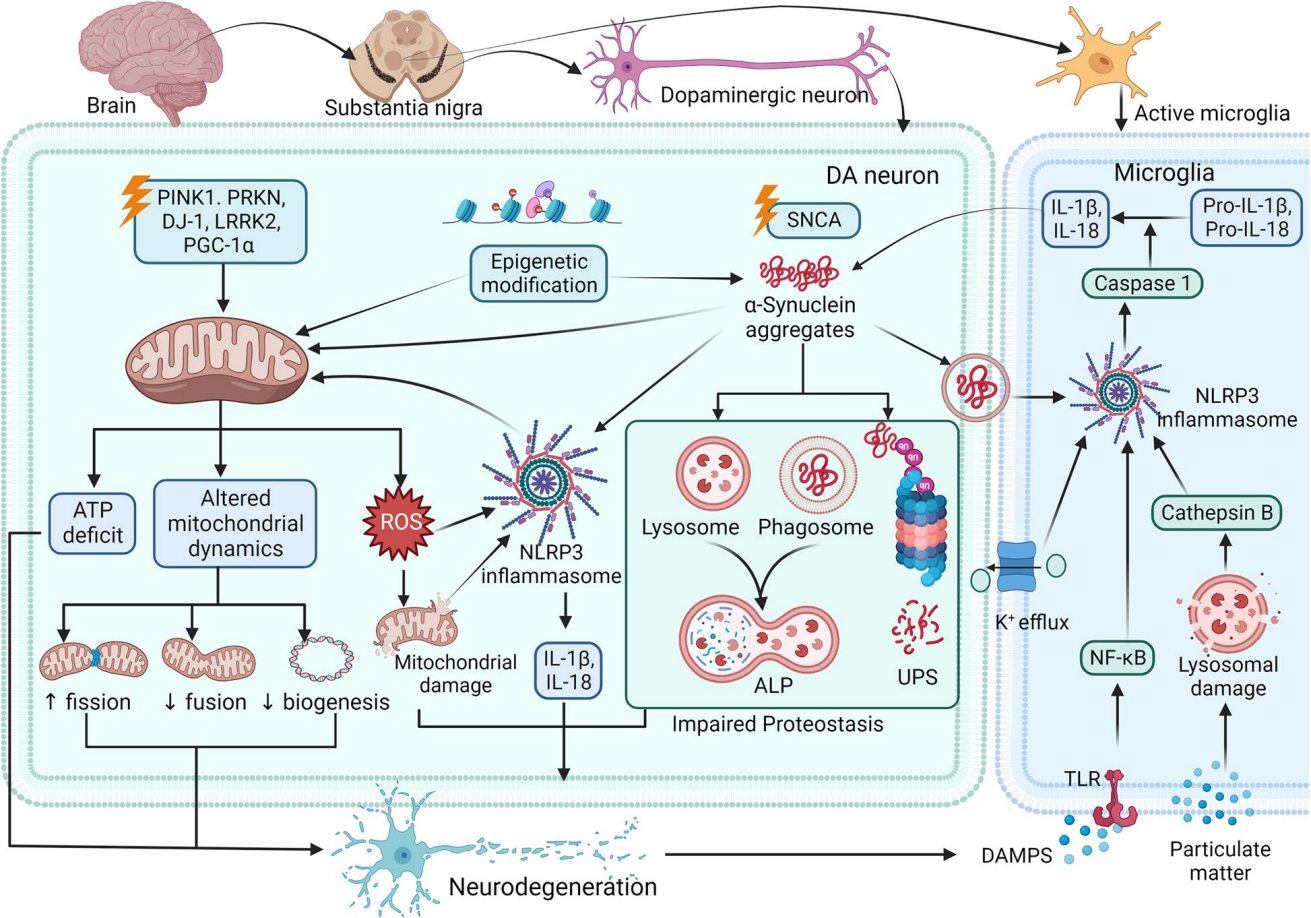
Overview of the pathogenesis of Parkinson's Disease. Various risk factors are involved in the pathogenesis of PD. Inherited as well as acquired gene mutations result in the generation of abnormal proteins. In the neuron, SNCA gene mutations encode an abnormal α-synuclein protein which forms aggregates and accumulates due to abnormal proteostasis mechanisms (UPS and ALP). Mutations in PINK1, PRKN, DJ-1, LRRK2, and PGC-1α are associated with mitochondrial dysfunction, resulting in ATP depletion, oxidative stress, and activation of neuroinflammatory pathways. Environmental chemicals like rotenone directly inhibit mitochondrial complex 1. The resulting DAMPs protein (damage-associated molecular patterns) and other pathologic events like K + efflux and lysosomal damage stimulate the NLRP3 inflammasome activation in the microglia, resulting in the generation of pro-inflammatory cytokines. These cytokines further propagate the inflammation, creating a vicious cycle that ultimately leads to the neuron's demise. DA: Dopaminergic, ALP: Autophagy-lysosomal pathway; UPS: Ubiquitin proteasome pathway; TLR: Toll-like receptor; ROS: Reactive oxygen species

Several mechanisms, such as neuroinflammation, protein mishandling, mitochondrial dysfunction, oxidative stress, and α-synucleinopathy, are involved in the pathogenesis of PD. An overview of the pathogenesis of PD is given in Fig. 1. The following section briefly describes some of the mechanisms.

### Pathological protein aggregation and α-synucleinopathy

In both sporadic and genetic cases of PD, the majority of the studies concluded the onset of Lewy bodies, the intracytoplasmic inclusions constituting aggregated α-syn proteins [18]. In genetic alterations, mutations of the *SNCA* gene [19], disruption of calcium homeostasis [20], impaired autophagy [21], oxidative stress, and other pathological conditions, the fibrillation process of α-syn starts with the formation of dimers from misfolded monomers, which further develop into small oligomeric substances stabilized by β-sheet-like interactions. Finally, high-molecular-weight insoluble protofibrils accumulate to build up as Lewy bodies [22]. Aggregated α-syn inhibits dopaminergic neurotransmission and reduces dopamine transporter levels. Mutant forms and-syn aggregates cause mitochondrial dysfunction, activate inflammatory pathways, and ultimately cause neurodegeneration [23].

### Impairment of protein degradation systems

The ubiquitin proteasomal system (UPS) and autophagy lysosomal pathways (ALP) are the major degradation pathways of abnormal proteins. Accumulating misfolded and aggregated proteins are commonly associated with neurodegenerative disorders and can be attributed to the impairment of these degradation pathways. The impairment of UPS and chaperone-mediated autophagy (CMA) may synergize with the accumulation of misfolded proteins, favoring subsequent fibrillation of α-syn. Endogenous α-syn is a substrate for CMA, and the posttranslational modification of α-syn has an inhibitory effect on this process [22]. Various animal and human studies have suggested the role of ROS in the pathogenesis of PD by intervening in the release of lysosomal proteases [24]. The proteasomal enzymes required for UPS were decreased in the SN region of PD patients. Additionally, mutations in autophagy-related genes such as *VPS35*, *LRRK2*, *PINK1*, and *PRKN* were strongly linked to familial PD. ALP is involved in the degradation of dysfunctional organelles, including mitochondria. In PD, mitophagy is severely affected and can be attributed to autosomal recessive genes such as *PINK1, DJ-1,* and *FBX07* [25, 26]*.*

### Mitochondrial dysfunction

Abnormal mitochondria were frequently found in the post-mortem samples of PD brains. Familial PD is strongly linked to gene mutations associated with mitochondrial functioning [27]. *PARKIN*, *PINK-1*, and *DJ-1* are responsible for mitochondrial quality control and dynamics, essential in repair mechanisms, the elimination of non-functional cell organelles, and mitochondrial biogenesis. Mutations in *PARKIN* and *PINK-1* disturb mitochondrial dynamics and cause the accumulation of dysfunctional mitochondria [28]. Additionally, impaired complex I activity may lead to free radical stress, increased radical scavenging activity, and glutamate excitotoxicity [29]. Administering various pesticides and other environmental toxins, such as rotenone [4, 5, 30, 31], 1-methyl-4-phenyl-1,2,3,6-tetrahydropyridine (MPTP) [32-34], and 1,1′-dimethyl-4,4′-5 bipyridinium (paraquat) [35-37], enables the establishment of PD models by replicating its pathological features. They are concentrated in DA neurons and inhibit complex I by causing systemic defects and leading to neurodegeneration by forming LB-like filamentous inclusions containing α-syn [38]. Mitochondrial dysfunction results in an energy crisis, generation of reactive oxygen species (ROS) and oxidative stress, activation of the immune system, and subsequent neuroinflammation resulting in the degeneration of neurons [39, 40].

### Neuroinflammation

Unlike neurons in the hippocampus or cortex, DA neurons display higher selectivity and sensitivity toward the damaging effects of inflammatory stimuli. Various animal and clinical studies have validated that neuroinflammation is involved in the progression of PD because of a compromise in DA neuron survival [41]. Neuroinflammatory responses occur due to glial activation and peripheral immune cell infiltration [42]. While oligomers of α-syn induce microglial responses through TLR2-mediated signaling, *LRRK2* elicits TNF-α and iNOS generation, regulating microglial activation and playing a vital role in peripheral inflammation [43]. Upon co-culture with microglia*, PARKIN* knockout (KO) species exhibited more significant inflammation due to the release of pro-inflammatory cytokines such as TNF-α, IL-6, and iNOS than wild-type neurons [44]. A better understanding of the neuroinflammatory mechanisms is required to provide a basis for future drug discoveries.

## CRISPR/Cas9 technology

CRISPR/Cas9 technology is preferred over gene editing techniques such as ZNF and TALEN because they offer several advantages. All gene editing can be completed within two weeks with high editing efficacy. The simultaneous editing of multiple sites and off-target sites can be easily predicted in the CRISPR/Cas9 system. When compared to ZFNs and TALENs, where proteins are recorded using DNA segments of 500–1500 bp length for every new target site, CRISPR Cas9 can be easily adapted to the target genomic sequence by subcloning the protospacer of gRNA [4, 5, 45].

The CRISPR/Cas9 system is divided into Class I (type I, III, and IV) and Class II (type II, V, and VI), depending on the design and role performed by the Cas proteins. While the Class I system utilizes multi-subunit Cas protein complexes, the Class II system uses a single Cas protein. Each type is characterized by a specific Cas9 protein enabling its differentiation. The type I system contains the Cas3 protein, which includes both DNase and helicase domains required to fragment the target. The type II system employs Cas1, Cas2, Cas4, or Cas9, whereas the Type III system constitutes Cas10 with an unclear role [46].

Among all the CRISPR/Cas9 systems, Type II CRISPR/Cas9 has a comparatively more straightforward structure, broadening its genetic engineering applications. It consists of three vital components: crRNA, tracrRNA combined to form a sgRNA and *Streptococcus pyogenes* originated-Cas9 protein. The sgRNA is created with crRNA, which locates the specific locus of the target DNA [47], and tracrRNA, a hairpin-like structure that binds to crRNA and forms an active complex with crRNA [48]. Cas9 protein is a 1368 amino acid containing DNA endonuclease (genetic scissor) that generates DSBs in the target DNA [46]. Through specific bioinformatic analysis, it was identified that Cas9 is composed of two regions: a gRNA binding recognition (REC) lobe with REC1 and REC2 subunits and a nuclease (NUC) lobe with a RuvC-like nuclease domain, His-Asn-His (HNH), a PAM-interacting (PI) domain, and a wedge (WED) domain [49]. The HNH domain fragments the DNA strand complementary to the 20-nucleotide sequence of crRNA. The RuvC domain targets the opposite strand of dsDNA 3 bp upstream of PAM sequences to cleave both strands of DNA. With the help of base-specific interactions, the PI domain interacts with the PAM region of DNA to enhance the DNA targeting specificity of Cas9. The WED domain is essential to identify sgRNA scaffolds to interact with the backbone of PAM [49-51]. The Cas9 protein has different variants based on the modifications in protein domains. While a Cas9 nickase (Cas9n) is created by mutating either HNH or RuvC, RNA-guided DNA binding protein is generated by mutating both domains. PAM is a 3-base pair (NGG) sequence present near the target site and is necessary for binding and Cas9-induced DNA editing [52].

**Fig. 2:**
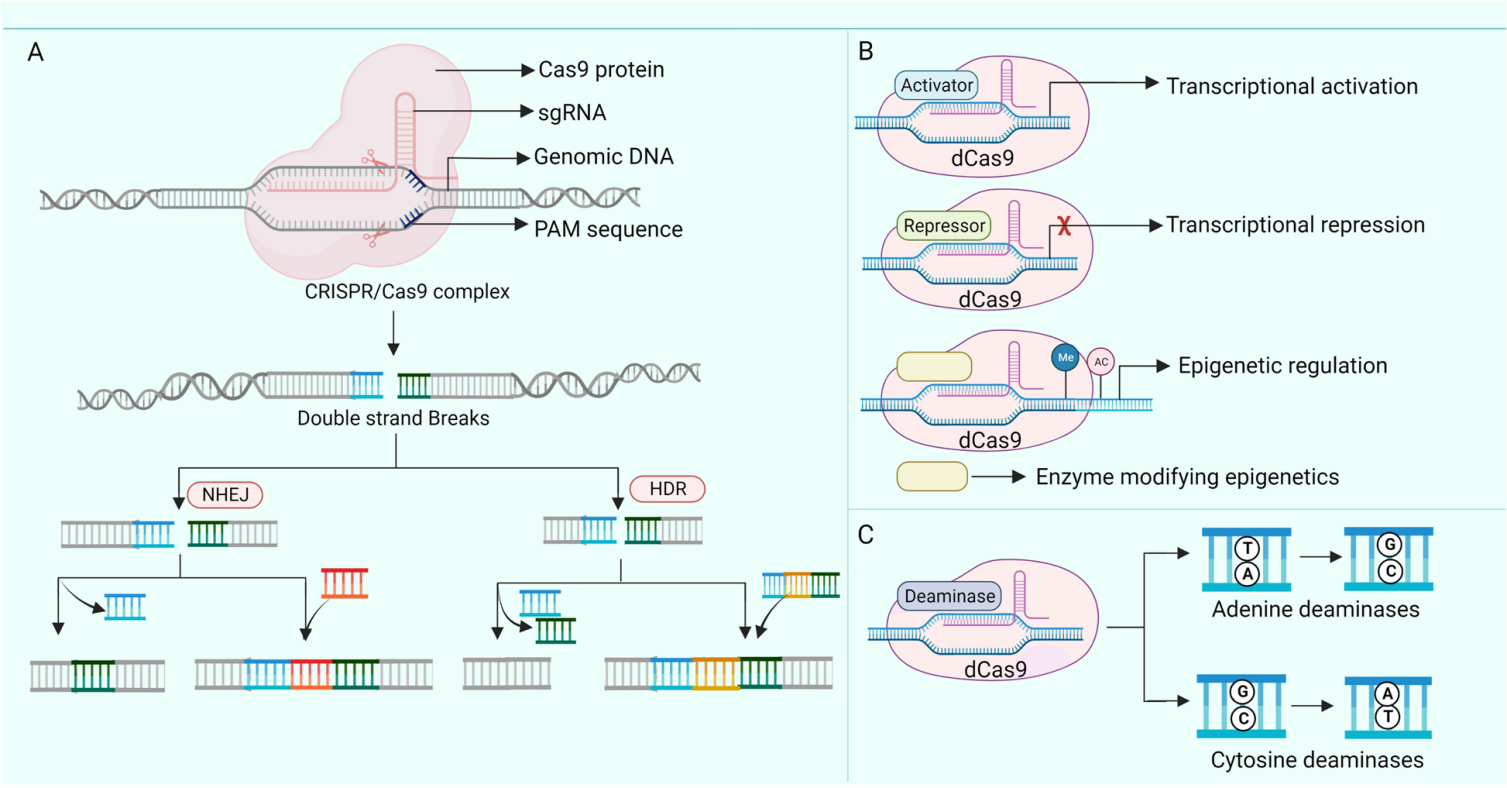
Mechanisms of CRISPR/Cas9 based gene editing. **A**. Schematic representation of mechanism of CRISPR/Cas9 based gene editing. Cas9 scans the DNA for the specific PAM sequence with the help of PAM interacting (PI) domain. Once the PAM sequence is identified sgRNA melts the target nucleotides upstream to PAM sequence and pairs them with crRNA. Then Cas9 cuts the target DNA 3 base pairs upstream of PAM sequence. Double strand breaks are repaired by Non homologous end joining (NHEJ) (majority) and Homology directed repair (HDR) resulting in random and precise deletions and insertions respectively **B**. CRISPR Cas9 based transcriptional regulation. Dead Cas9 (dCas9) is fused with specific transcription regulatory domains. Upon binding to the target DNA site, dCas9- regulatory domain complex recruits the corresponding gene activators/repressors to carry out the regulatory function **C**. CRISPR/Cas9 based base editing. dCas9 or Cas9 nickase (nCas9) are used for base editing. Cytosine base editors deaminate cytosine to uracil which is recognised by the DNA repair mechanisms and is substituted with Adenosine, whereas Adenosine base editors deaminate Adenosine to inosine which is substituted with Guanine by repair mechanisms

The mechanism of CRISPR/Cas9 was demonstrated by using a combination of structural studies and in vitro assays. In short, CRISPR/Cas9 functions by the following steps in sequence: Initially, the Cas9 endonuclease is expressed, followed by creating a 20-nucleotide sequence of sgRNA complementary to the target DNA. Finally, Cas9 will cleave near the PAM site proximate to the 3′ end of the target region [53]. When not bound to sgRNA, Cas9 exists in an autoinhibitory conformation due to the blockage of active sites of HNH domains by the RuvC domain. It is converted to a DNA-recognition-competent conformation upon attachment of sgRNA to create a central channel between the two lobes for DNA binding [50]. After searching for the PAM in the target DNA through three-dimensional diffusion, the sgRNA-Cas9 complex binds to the PAM via the PI domain. DNA strand separation enables the formation of a sgRNA-DNA duplex starting from the PAM-proximal region [54]. Strand unwinding and separation occur only when there are structural similarities between the gRNA and target DNA [55]. A strong association between sgRNA and DNA further accelerates DNA strand separation until the PAM-proximal region forms a complete R loop, which instigates another conformational change in the HNH domain to induce the formation of DNA breaks by activating the nuclease activity of both RuvC and HNH domains [56, 57]. Cas9 is tightly bound to the cleaved target DNA until other cellular factors detach the enzyme for recycling [47]. The DSBs that Cas9 creates are joined either through NHEJ or HDR (Fig. 2). NHEJ is highly prone to errors because it ligates the fragmented end of the target DNA to form random DNA indels. In contrast, HDR utilizes a precise repair mechanism by adding a homologous donor DNA template to restore the damage [58].

## Application of the CRISPR/Cas9 system in PD

**Fig. 3:**
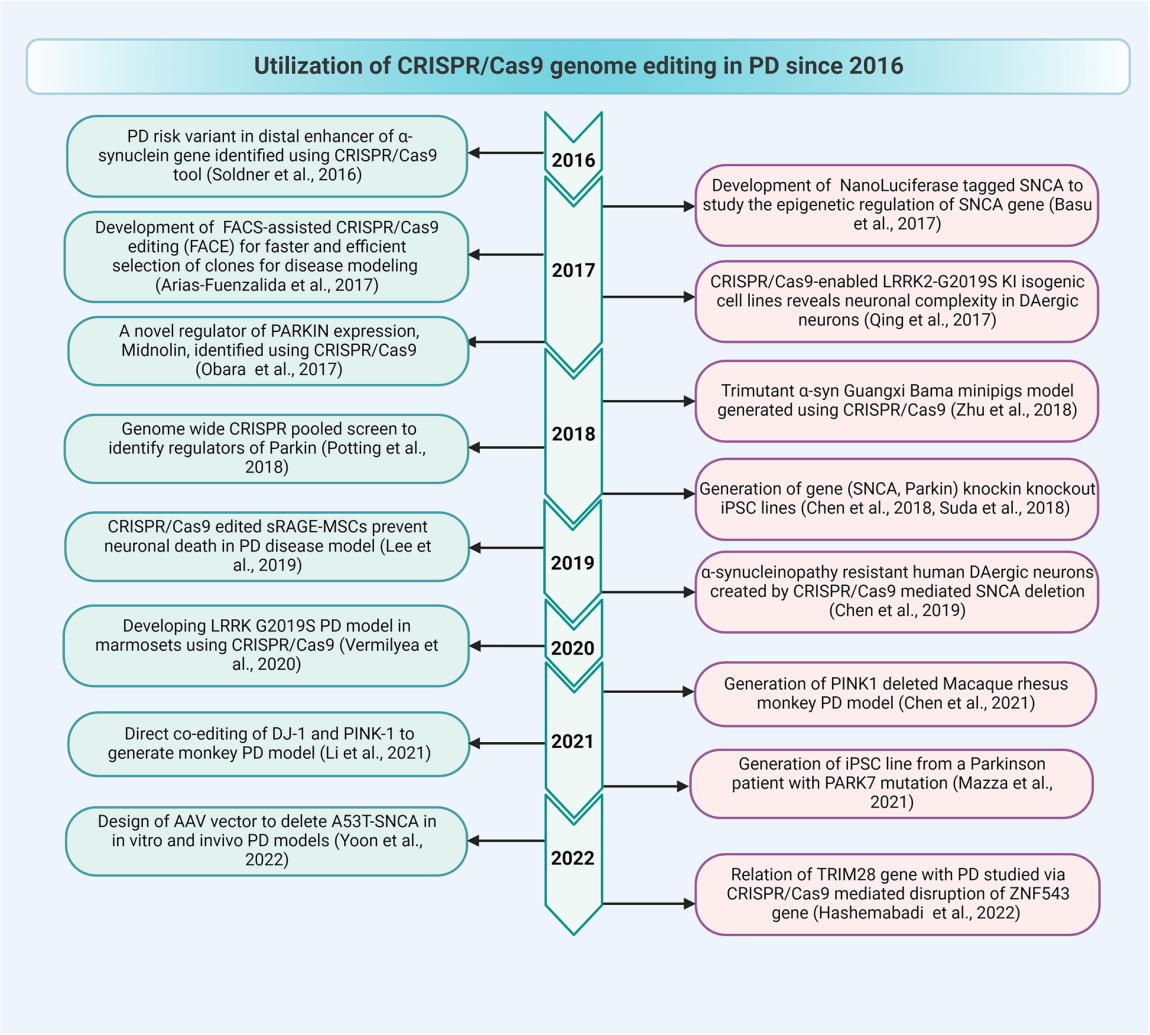
Timeline for application of CRISPR/Cas9 tool in PD research

**Fig. 4:**
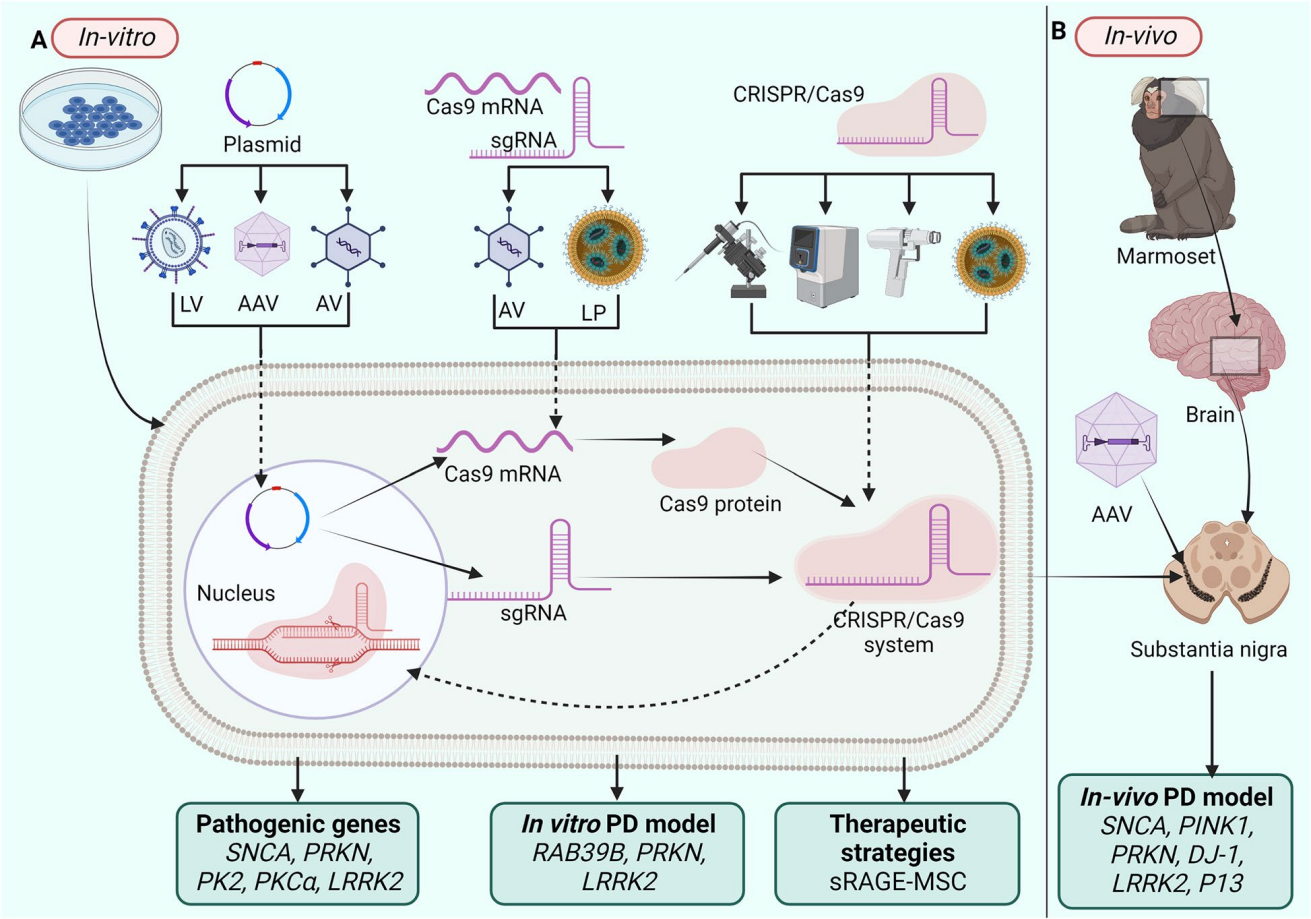
Applications of CRISPR Cas9 tool in Parkinson's Disease. **A**. In-vitro application of CRISPR/Cas9 tool. Plasmids, Cas9 mRNA, SgRNA, or whole Ribonucleoprotein can be delivered into the biological systems using various methods. In-vitro applications in PD include the identification of pathogenic genes, model development, and the development of therapeutic strategies. **B**. In vivo applications of the CRISPR/Cas9 tool includes PD model development and screening of therapeutic strategies

CRISPR/Cas9 induces alterations and gene expression, enabling researchers to explore and treat neurodegenerative disorders, especially PD, which has limited disease-modifying approaches. The timeline of applications of the CRISPR/Cas9 tool in PD is shown in Fig. 3. Various implications of the CRISPR/Cas9 tool in PD are discussed in the following section and depicted in Fig. 4.

### Developing disease models

CRISPR/Cas9 is the most efficient approach to perform genetic manipulation to create disease models that mimic the genetic and phenotypic phenotypes of PD [59]. Because of the neurophysiological and neuroanatomical similarities between minipigs and humans, minipigs are valuable tools for modeling neurodegenerative diseases. CRISPR/Cas9 combined with somatic cell nuclear transfer (SNCT) was used to generate Guangxi Bama minipigs expressing mutations in *SNCA*, namely, E46K, H50Q, and G51D, to establish PD. A total of eight SCNT-derived Guangxi Bama minipigs with the desired *SNCA* mutations were integrated into the genome. The overexpression of α-syn at the transcriptional level was examined by DNA sequencing. The immunohistochemical analysis confirmed α-syn immune-positive pathology and nigrostriatal neuronal loss [60]. A PD pig model using Bama miniature pigs was created by co-administering CRISPR/Cas9 to simultaneously target three distinct genomic loci, *PARKIN*, *DJ-1*, and *PINK1* genes. Despite using multiple sgRNAs, whole genome sequencing suggested that the incidence of off-target events was low, demonstrating the efficacy and power of CRISPR Cas9 [56, 57].

Isogenic loss-of-function models of early-onset autosomal recessive PD (PARKIN^−/−^, ATP13A2^−/−^ and DJ-1^−/−^) were created to correlate the dysfunction of *PARKIN, ATP13A2,* and *DJ-1* with the neurodegeneration of nigral dopaminergic neurons in human pluripotent stem cells. PD gene loss was associated with depletion of mitochondrial proteins, increased oxidative stress, altered lysosomal functions, cell death of early TH^+^ neurons, and higher expression of the VTA marker CALB1 in *PARKIN* organoids, indicating severe damage to A9-like neurons [61]. An SH-SY5Y cell model showing cellular aging and PD was generated by instantly removing telomeres via CRISPR Cas9 technology, impairing mitochondrial respiration and cell viability. Approximately 50%, 80%, and 40% decrease in *PGC-1α*, *NRF1*, and *PARKIN* genes, respectively, all involved in mitochondrial biogenesis and quality control, were observed. In response to telomere removal, DNA damage sensors such as p53 and *PARP1* were increased. These findings demonstrated that CRISPR Cas9 is being efficiently utilized to model PD(H. [62, 63].

### Targeting pathological genes

#### PARKIN

*PARKIN*, an E3 ubiquitin ligase, regulates the selective removal of dysfunctional and unproductive mitochondria, thus fine-tuning the mitochondrial network and conserving energy metabolism. *PARKIN*, combined with *PINK1*, reduces impaired mitochondria via mitophagy [64]. Previously, RNAi screens were used to detect regulators of *PARKIN*-mediated mitophagy but did not provide satisfactory results. A phenotypic genome-wide CRISPR Cas9 pooled screen was used to identify the molecules that regulate *PARKIN*. Cells expressing the *PARKIN* regulator system were utilized to detect 53 positive and negative factors involved in mediating *PARKIN* function by calculating its kinetics through phosphoS65-ubiquitin (pUb) accumulation. The functional analysis of the transcriptional repressor THAP11 highlighted the impact of endogenous PARKIN-level regulation on pUb accumulation in multiple cell types, including postmitotic iPSC-derived iNGN2 neurons [65].

#### P13

*p13* is a mitochondrial matrix protein essential to modulate PD pathogenesis in familial and toxin-induced PD cases and can be considered a therapeutic target for PD. It exerts its effects by binding to the electron transport chain complexes I and V named NDUFAB1 and ATPAF2, and an increase in p13 promotes the assembly of complex I. An experiment was performed to test whether p13 levels were altered in PD models; a reduction in p13 levels occurred due to mitochondrial dysfunction and cellular injury in the rotenone-induced PD model and MPTP-treated mice. p13 KO mice were created through CRISPR/Cas9, which showed no motor deficits or dopaminergic neuronal loss following MPTP treatment due to the assemblage of Complex I more readily upon toxin exposure. Mitochondrial functions were conserved, and neuronal homeostasis was maintained [66, 67].

#### Prokineticin 2 (PK2)

In the brain, PK2 enables neurogenesis by promoting the shift of progenitor cells from the subventricular zone [68]. It promotes mitochondrial biogenesis and activates the ERK and Akt signaling pathways, promoting cell survival [69, 70]. A study revealed that *PK2* mRNA is overexpressed during dopaminergic cell death triggered by TNF-α indicating that *PK2* is a promising signaling mediator synthesized during dopaminergic degeneration [71]. By increasing the levels of *PGC-1α* and *BCL2* and restoring the loss in ATP production, overexpression of PK2 restores mitochondrial biogenesis. The CRISPR Cas9 system was used to knock out *PK2*, resulting in increased susceptibility of the neurons to cell death upon MPP^+^-induced toxicity. This effect was reversed upon the addition of recombinant *PK2,* which activates the ERK and Akt signaling pathways [69, 70]. Furthermore, the KO of *PKR1* blocked the efficiency of *PK2* in activating alternative A2 astrocytes. Both mechanisms were confirmed to be associated with mitochondrial dysfunction and neuroinflammation through CRISPR Cas9-mediated gene editing [72].

#### Protein Kinase Cδ (PKCδ)

Increased kinase concentrations are found in SNpc dopaminergic neurons, primary dopaminergic cell lines, and dopaminergic cultures [73]. The proteolytic fragmentation of *PKδ* triggers neuronal cell death through Caspase-3 in dopaminergic cells. Additionally, inflammatory and immune responses are mediated through the activation of PKδ(Γορδον,Σινγη,εταλ.,2016). The PKδ KO in N27 dopaminergic cells showed reduced endosulfan-induced apoptosis. Efficient molecules targeting autophagy by targeting *PKδ* can act as therapeutic agents [74].

### Identification of potential drug targets

Target identification is vital in probing small molecules and drug discovery; it ensures that the process is carried out in a disease-specific manner. Upon the binding of the drugs to the potential target, they selectively modulate the function of the target proteins. Two novel drug targets were discovered through CRISPR/Cas9 that significantly alter the disease pathology, i.e., p62 and midnolin.

Autophagic pathway impairment is crucial in PD, as it aggregates abnormal proteins and damages functional organelles. It was found that S-nitrosylation of the adapter protein p62 inhibits autophagic flux and causes the build-up of misfolded proteins, subsequently promoting the concentration of α-synuclein and the spread of the disease. To test this hypothesis, cell-based models were generated through neurogenic cell lines and CRISPR Cas9 technology. It was concluded that the PD-diseased cell lines exhibited greater p62 levels than the wild-type isogenic control cell DA neurons [75]. A new regulator of *PARKIN* expression, midnolin (*MIDN*), was found to be associated with the pathology of PD. *MIDN* expression is primarily seen in the nucleus and intracellular vesicle membranes of PC12 cells and is promoted by NGF and cAMP signaling. Inhibition of NGF-induced neuronal outgrowth increased the accumulation of misfolded proteins, and loss of parkin expression was observed when *MIDN* was knocked out. Additionally, approximately 10.5% of sporadic PD patients lacked a copy of the *MIDN* gene, which is not exhibited in normal individuals, indicating a strong link between *MIDN* loss and PD. Hence, drug molecules targeting the increase in this novel protein can be considered to alleviate PD symptoms [76].

### Transcriptional regulation

Cas9-based transcriptional regulation is a vital tool for basic science and biotechnological applications, especially screening for gene functions and novel gene regulatory circuits and decoding the pathological mechanisms in PD [77]. Although the *ZNF543* gene is considered relevant to familial forms of PD, there is no evidence detailing the mechanisms involved. The genetic location of the *ZNF543* gene is that of *TRIM28,* which promotes the accumulation and toxicity of α-syn. It was assumed that *ZNF543* might act as a transcriptional activator for *TRIM28* gene expression. An efficient *ZNF543* gene transcriptional activation model was generated using CRISPR/Cas9 gene editing in human neuroblastoma cell lines. The results indicated an increase in *TRIM28* gene expression, indicating the association of *ZNF543* with *TRIM28* in mediating parkinsonism [78]. In another study, essential histone posttranslational modifications were identified, i.e., transcriptional promoting marks H3K4me3 and H3K27ac and repressive mark H3K27me3 of the SNCA promoter using the ENCODE database. H3K27me3, which is more significantly increased at the SNCA promoter of the SNpc region of PD patients, is involved in regulating the concentration of α-syn. To validate this, a CRISPR/dCas9-based locus-specific H3K4me3 demethylating system was developed by acquiring the catalytic domain of JARIDIA into the SNCA promoter to obtain CRISPR/dCas9SunTag-JARID1A. A decline in H3K4me3 at the SNCA promoter was linked with a decrease in α-syn concentration in the SH-SY5Y cell line and idiopathic PD-iPSC-derived DA neurons. Thus, altering histone modifications can play a crucial role in alleviating PD pathology by minimizing α-syn levels [79]. In general, *THAP11* is a negative regulator of the PARKIN protein. THAP11-mediated transcriptional repression of *PARK2* and pUb accumulation was addressed in hiPSCs. carrying iNGN2. iNGN2 THAP11^±^ neurons generated using CRISPR/Cas9 exhibited a considerable increase in PARKIN protein levels, revealing *THAP11*-mediated transcriptional repression in neurons, where an increase in PARKIN levels limits the incidence of early damage-induced pUb accumulation [65]. The correlation of transcriptional regulation of the *SNCA* gene with PD pathology was confirmed by a stable cell line with a NanoLuc luciferase reporter tagged at the 3′ end using CRISPR/Cas9 gene editing. The luciferase reporter efficiently monitored the transcriptional regulation of SNCA after treatment with DNA methyltransferase 1 (DNMT1) inhibitors and histone deacetylase (HDAC) inhibitors [80].

## Application of stem cell technology in PD

### Induced pluripotent stem cells (iPSCs)

iPSCs are derived from adult somatic cells and are pluripotent stem cells that can be genetically transformed into an embryonic stem (ES) cell-like state upon artificial expression of genes and factors necessary to maintain the defining properties of ES cells. iPSCs have pluripotent and self-renewal properties. In 2006, Dr. Yamanaka and his team identified that pluripotent cells could be generated from fibroblasts by expressing defined embryonic transcription factors such as *OCT4*, *SOX2*, *KLF4*, and *MYC*. Currently, iPSCs can also be developed from neural stem cells, liver and stomach cells, and peripheral blood cells [81]. iPSCs are advantageous, as they avoid immune rejection because stem cells can be created from the same patient who needs a transplant. Ethical issues can be avoided since stem cells are generated from a willing adult. Cells of unlimited proliferation with all three germ layers are produced, with their only limitation being tumorigenicity [82].

By observing the disease-related phenotypes in PD iPSC-derived neurons, the patient-specific molecular mechanism of disease, the effect of the environmental factors, and potential therapeutic candidates can be assessed. Dopaminergic neurons were derived from PD patient iPSCs exhibiting triplication of the *SNCA* gene. These cells overexpressed α-synuclein and oxidative stress markers. PD mutations are more capable of intrinsically affecting normal cell function, and further deriving neurons from iPSCs helps obtain a better overview of the complex polygenic background [83]. The role of overexpressed α-synuclein in compromising the process of phagocytosis was established using iPSC-derived macrophages. Initially, iPSCs were generated from early-onset PD patients with SNCA triplication mutations and differentiated into PSC macrophages (pMacs). Although pMacs degrade α-synuclein by actin-dependent and actin-independent pathway-mediated uptake, high levels of endogenous α-synuclein were found to compromise this ability [84]. iPSCs facilitated the modeling of young-onset PD (YOPD) to reveal a significant marker of disease and novel therapeutic compounds. iPSCs were extracted from YOPD patients with no known mutations and differentiated into neurons. Elevation of α-synuclein and phosphorylated protein kinase Cα and decreased expression of lysosomal membrane proteins such as *LAMP1* were observed. A specific phorbol ester, PEP005, was tested for its efficiency in activating lysosomal function. This compound enhanced the *LAMP1* concentration and reduced the α-syn and Cα concentrations, indicating that phorbol esters can be considered a therapeutic option for PD [85]. It is known that mitochondrial damage has a crucial role in the pathology of PD. iPSC-derived neurons from GBA-PD patients and a *GBA* KO- *Drosophila* model that exhibited stress responses, mitochondrial death, and alterations in NAD^+^ metabolism were used to establish this link. The maintenance of the NAD^+^ pool and synthesis of NAD^+^ precursors are required to reverse pathological symptoms. This is facilitated by administering nicotinamide ribonucleotide, which significantly alleviates mitochondrial function and prevents age-related neuronal loss, suggesting its neuroprotective action [86].

Similarly, the pharmacological rescue of mitochondrial deficits by CoQ10, rapamycin, and the *LRRK2* kinase inhibitor GW05074 was successful in iPSC-derived neural cells from individuals carrying *PINK1* and *LRRK2* mutations. It was evaluated using parameters such as the generation of ROS, mitochondrial respiration, proton leakage, and intraneuronal movement of mitochondria [87]. iPSCs were utilized for a novel application of establishing dopaminergic neurons with transcriptional profiles similar to those of neurons isolated from PD patients. iPSC-derived human dopaminergic neurons (DaNs) were purified by using an intracellular marker, TH, and the transcriptomic profiles of these neurons matched those of mature post-mortem DaNs, indicating that these cells can act as an appropriate in vitro disease model for PD [88].

### Embryonic stem cells (ESCs)

ESCs are abundantly present in the inner cell mass of the human blastocyst, a preliminary phase of the evolving embryo from the 4^th^ to the 7^th^-day post-fertilization [89]. They are mainly characterized by an exceptional ability to renew and can form the three embryonic germ layer lineages upon induction of differentiation [82]. Their clinical application is limited because of two significant hurdles: tumor formation and immune rejection. Tumor formation can be avoided by differentiating the cells into lesser malignant variants, such as neurons, muscle, and liver cells. Immunosuppressants can suppress immune rejection, or ESCs could be made less reactive through genetic engineering by removing the surface antigens responsible for immune reactions [89].

Various strategies, such as replacing dysfunctional dopaminergic neurons with hESCs in SNpc patients with PD; utilizing hESCs to transfer neurotrophic factors, cytokines, chemokines, or growth factors to rescue the degenerating tissue; transplanting hESCs that express transcription factors involved in PD phenotypes; stimulating neural stem cell proliferation and repair by transplantation of genetically engineered hESCs; and stimulating the synthesis of endogenous factors that restore the injured brain, have been developed to apply ESCs in treating PD [90].

It has been reported that ESCs have great potential to generate midbrain precursors and dopamine neurons. ESC-derived TH^+^ cells can release dopamine, create functional synaptic connections, extend axons into the host striatum, and modify pharmacologically induced behavior. Even at low cell densities and doses, ESCs differentiated into mesencephalic TH^+^ neurons in the rat striatum. They were beneficial in the functional recovery of amphetamine-induced motor asymmetry in a rat model of PD [91]. Regionally specific neural progenitor cells and active neurons were generated from hESCs under defined conditions. Using the *GSK3* inhibitor CT99021, dose-dependent activation of canonical *WNT* signaling was established to simulate the positional patterning of the neural tubes in differentiating hESCs. These positionally specified progenitors formed neuron-rich grafts ten days after transplantation. Similarly, patterned cultures of ventral mesencephalic fate were transplanted into an animal model of PD to develop into a large number of functional dopaminergic neurons [92].

When mESCs differentiate into dopaminergic neurons, the primary issue is the development of a teratoma. Hence, in a study, ESCs, before being transplanted into the affected PD mice, were treated with mitomycin C (MMC, a DNA alkylating agent) to prevent the emergence of a tumor. Teratoma formation was successfully prevented after its administration into nude mice. Later, intrastriatal injection of MMC-treated ESCs improved motor function without tumor formation in 6-OHDA-lesioned mice [93]. Cripto is a coreceptor that binds to Nodal and ALK-4 receptors and plays a vital role in stem cell functioning. It was demonstrated that inhibition of cryptocytes is associated with increased differentiation of mESCs into dopaminergic neurons and is thus a potent therapy for PD. A Cripto blocking peptide (CBP) that interferes with the Cripto/ALK-4 receptor interaction was developed. The mESCs were treated with CBP to establish CBP-treated mESCs, which on transplantation, enhanced functional recovery and decreased tumor formation in the striatum of PD rats [94].

### Mesenchymal stem cells (MSCs)

With mesodermal origin, MSCs can differentiate into osteoblasts, chondrocytes, adipocytes, and myocytes [82]. They show plasticity and markers on their cell exteriors, such as *CD105*, *CD90*, *CD73*, *CD29*, and *CD44*. These cells are sequestered from amniotic fluid, endometrium, adipose tissue, peripheral blood, placenta, and synovial fluid, among others [95]. They are applied therapeutically for their potential to drift toward the area of degeneration, remarkable transforming ability, and low immunogenicity.

Treating MSCs in the neurodegenerative regions of the brain is considered a potential therapeutic strategy to overcome PD. Certain studies have indicated that compromised BBB infiltration could be a reason behind the pathogenesis of PD. Thus, MSCs were administered systemically to the MPTP-induced PD model. MSCs repaired the BBB, resisted microglial activation, and facilitated the release of TGF-β1 [96]. Intranasal administration of human-endometrial-derived stem cells (hEDSCs) was also found to improve behavioral deficits due to the migration of hEDSCs into the SNpc region, which was further supported by an increase in the concentration of the dopaminergic neuronal markers Nestin and TH [97].

MSCs can also deliver a therapeutic gene to facilitate gene therapy in PD. In a study, MSCs were transduced with the *TH* gene through AAV and transplanted into the striatum of PD rats exhibiting asymmetric rotation upon apomorphine administration. Interestingly, TH-expressing MSCs were detected to differentiate into fibroblast colony-forming units rapidly. Furthermore, they significantly decreased apomorphine-induced asymmetric rotation and exhibited greater dopamine levels [98]. Similarly, glial cell line-derived neurotrophic factor (GDNF) was engineered to be incorporated into MSCs and delivered to disease-affected neurons. The proliferation capacity and therapeutic efficacy of human primary adipose-derived MSCs (hAMSCs) were increased by preexposure to GDNF. Intracranial injection of hAMSCs-GDNF in 6-OHDA lesioned mice enabled greater performance on behavioral tests, increased TH levels, and larger graft volumes [99].

To overcome the low survival rate and tumor-forming capacity of MSCs and deliver therapeutic molecules, exosomes derived from MSCs are utilized as an alternative to repair degeneration in PD models. Exosomes isolated from human umbilical cord mesenchymal stem cells (hucMSCs) reached the substantia nigra through the BBB to reduce dopaminergic neuron loss and elevate dopamine levels by promoting autophagy. The uptake of exosomes by 6-OHDA-stimulated SH-SY5Y cells was confirmed by labeling them with PKH26 [100, 101]. Bone marrow-derived MSC-derived exosomes containing Gli1 promoted cell proliferation, inhibited apoptosis in MPP^+^-treated SH-SY5Y cells, ameliorated inflammatory damage, alleviated microglial activation, and inhibited Sp-1-induced *LRRK2* activation and neuronal injury [102]. To enable efficient production of designer exosomes that allow specific mRNA packaging and delivery of mRNA into the cytoplasm of the targeted cells, a set of exosomal transfers into cells through (EXOtic) devices is performed. These genetically engineered cells are transplanted into diseased mice to deliver therapeutic catalase mRNA to the brain to facilitate the elimination of neurotoxicity and neuroinflammation in PD models [103].

Currently, three clinical trials are in the pipeline to evaluate the safety, efficacy, and feasibility of MSC-based therapy in PD patients. The first trial plans to perform pilot phase I studies using allogenic bone-marrow-derived MSCs from idiopathic PD patients (NCT02611167). A subsequent study intended to utilize umbilical cord-derived MSCs through an IV infusion (NCT03550183). The NCT03684122 trial predicts to confirm the differentiation of allogenic MSCs into NSCs and rescue the motor and nonmotor symptoms of PD.

### Neural stem cells (NSCs)

NSCs are present in the subgranular zone (SGZ) of the dentate gyrus in the hippocampus and the subependymal zone (SEZ) of the lateral ventricles. NSCs divide continuously until they lose their stemness, then differentiate into astrocytes, neurons, and oligodendrocytes. While studying the pathology of PD, it was found that there is a strong connection between the altered generation of NSCs and functional DA neurons. The transcranial injection of NSCs into the diseased tissues determines their viability, propagation, differentiation, and neuroregeneration. Sometimes, NSCs are integrated with ECs to enhance the rate of survival, differentiation, and neuronal proliferation [104]. NSCs restore damaged DA neurons by initiating neuron regeneration through differentiation reactions. NSCs can be isolated from adult and fetal CNS tissues, while adult NSCs have a lower differentiation potential than fetal NSCs [82]. To restore motor function, *Pitx3* was overexpressed in NSCs and transplanted into 6-OHDA-lesioned PD rats. The number of dopaminergic neurons within the peripheral area of the graft increased [105]. NSCs can deliver neuroprotective molecules such as GDNF to prompt the reversal of PD pathology. NSCs were engineered to release GDNF into degenerated dopaminergic neurons in the SNpc by giving rise to neurons, astrocytes, and oligodendrocytes that expressed stable levels of GDNF for approximately four months and decreased behavioral impairment in PD-affected mice [106].

The function of nigral dopaminergic neurons can be regulated by enhancing the expression of the proteins that induce a neuroprotective action. Persephin (PSP), a neurotrophic factor of the GDNF family, amplifies the survival and neurogenesis of midbrain dopaminergic neurons. NSCs were engineered to overexpress PSP protein for approximately three months within the striatum. PSP-overexpressing cells improved dopamine-dependent behavioral parameters and suppressed the loss of dopamine neurons [107]. Insulin-like growth factor 1 (IGF-1) is also known to show neuroprotective activity in many disease models. Hence, neural progenitor cells expressing *IGF-1* alleviated amphetamine-induced rotational disturbances and dopaminergic neuronal deficits within seven days of transplantation into a 6-OHDA-induced lesion model [108].

Synergistic effects can be achieved by combining NSCs with therapeutic agents to overcome neurodegeneration in PD. Fasudil, a Rho kinase inhibitor, was administered simultaneously with NSCs in a PD disease model to display more potent anti-inflammatory and antioxidant effects and homeostasis of NMDA and AMPA receptors, indicating that this combination can be considered a promising cell-based therapy [109]. Similarly, lithium chloride (LiCl) treatment was also found to promote the neuronal differentiation of NSCs, mediated through the WNT signaling pathway. The beneficial effect of LiCl was demonstrated using a 6-OHDA-induced PD model through enhanced motor function, learning, and memory supported by increased spine density [110].

Overall, studies using the CRISPR/Cas9 tool to modulate neurogenic transcription factors in NSCs are highly beneficial and expected to yield interesting results in PD research.

## CRISPR/Cas9 genome editing in PD stem cells: a unique strategy

Though CRISPR/Cas9 and stem cells are being individually tested to treat PD, when combined, these techniques can potentially transform the field of regenerative medicine. They are being used to identify novel pathways implicated in PD pathogenesis and develop therapeutic strategies against it as a deeper understanding of the genotypic and phenotypic parameters of the disease is attained.

### Developing disease models

#### iPSCs

iPSCs derived from the neurons of PD patients are used to model PD, confirm the pathophysiology in DA neurons, screen potent therapeutic substances, and understand their effect on iPSC from PD patients. In a study, heterozygous missense *A30P* and *A53T* mutations were introduced into the *SNCA* gene in healthy iPSCs using FACS-mediated CRISPR/Cas9 to generate the PD cellular model. The altered *SNCA* mutant iPSCs exhibited a remarkable decline in maximal respiration, basal respiration, nonmitochondrial respiration, proton leakage, and ATP production [111]. These traits were comparable with the features of the formerly described *SNCA.* The *A30P* mutant differentiated neurons proved the relevance of FACS-mediated CRISPR/Cas9 gene editing for creating disease-relevant models [112].

Along with *SNCA*, the genetically dominant *p. G2019S* mutation of *LRRK2* induces α-syn toxicity and alters neuronal homeostasis due to overexpression of its kinase activity. CRISPR/Cas9 nuclease and piggyBac transposase system induced heterozygous *LRRK2-G2019S* point mutation into hiPSCs. A compromise in neuronal complexity was identified by reduced TH-positive neurons and average branch number in the edited hiPSCs [113]. In another study, *LRRK*-mutant iPSCs also showed increased Caspase-3 and ROS activation, autophagy disruption, reduced neuronal viability and decreased neuronal complexity. Similarly, two *LRRK2* homozygous KO hiPSCs were generated using CRISPR/Cas9 without altering the morphology, gene expression, and differentiation potential. They are beneficial to understand the importance of *LRRK2* in PD and innate immunity [100, 101].

Additionally, homozygous *PRKN*, *PINK1*, and *PINK1/PRKN* double KO iPSCs and *PARK7-*mutated Gibco iPSCs, a type of viral integration-free iPSCs, were generated [114]. These iPSCs are considered valuable tools for examining *DJ-1*-linked function in translational PD research. The research aims to create more PD-specific iPSC-based models for late-onset PD, which could replicate the motor and nonmotor symptoms in animal and cell disease models [115]. The association of endogenous ghrelin receptors (GHSRs) with PD was confirmed using CRISPR-generated PARKIN mutant iPSCs. A downregulation of GHSRs and corresponding mRNA was observed in mutant iPSCs compared to the control groups [116].

#### Using ESCs

ESCs act as vital disease models to understand the role of specific genes in PD-based neurodegeneration. Recently, the mutations in *DNAJC6* encoding HSP40 auxilin were found to induce early-onset PD. Isogenic hESCs with detrimental mutations in intron 6 to exon 7 of the *DNAJC6* locus were generated using the CRISPR/Cas9 tool. Excessive α-synuclein accumulation, dopaminergic neurodegeneration, mitochondrial and lysosomal dysfunction, impaired *WNT-LMX1A* autoregulation, defects in general neurogenesis, and increased neuronal misfiring were observed, validating the link between *DNAJC6* and PD pathogenesis [117].

PD intensifying regions, i.e., single nucleotide polymorphisms (SNPs), *rs356168* and *rs3756054*, were deleted from hESCs using CRISPR/Cas9 system. One of these alleles was incorporated into heterozygous cells and allowed to differentiate into neural precursors and neurons. The *SNCA* mRNA transcripts in these genetically altered cells showed a remarkable increase in *SNCA* expression levels, suggesting the role of these alleles in PD pathology [118]. Moreover, *RAB39B*, *PARP1*, and *KEAP1* KO isogenic hESCs were generated using CRISPR/Cas9 to model PD as they expressed normal karyotype, stem cell markers, and retained differentiation potential [62, 63, 119, 120].

#### Using NSCs

The ability to extract and study NSCs from transgenic, mutant, knockout, and disease model mice has made them an excellent model. NSCs were generated from iPSCs of early-onset diseased patients containing either a point mutation (*A53T*) or triple replication at the α-synuclein/*SNCA* gene locus (S3). The correction of the mutated gene *A53T* and *SNCA* KD using CRISPR/Cas9 was performed to obtain *A53T*/Corr and S3/KD, respectively. Later, neurospheres were generated from *A53T*/Corr and S3/KD NSCs to replicate the brain tissue microenvironment. These neurospheres were used to study the age-dependent increase in α-synuclein, examine targets, and explore the effect of possible small molecule therapeutics. An age-dependent accumulation of pS129 α-syn was observed in these neurospheres. Additionally, the neuroprotective effect of CAY10566, a stearoyl CoA desaturase inhibitor, via action on the abnormal fatty acid synthesis and α-syn accumulation was also reported through these NSC-derived neurospheres [121].

### Developing therapeutic strategies

Mutations in the *GBA1* gene influences glucocerebrosidase (GCase) enzyme and poses a risk factor in PD. iPSC-derived dopaminergic neurons lacking DJ-1 were generated from two patients with idiopathic PD. A novel modulator of GCase named S-181 increased the wild-type GCase enzymatic activity and rescued the accumulation of oxidized dopamine in these genetically engineered neurons. The activation of wild-type GCase by small-molecule modulators could be considered an advanced pharmacological intervention for treating familial and sporadic PD forms.

ESCs have been employed to create synucleinopathy-resistant human dopaminergic neurons, a novel mechanism explored to overcome PD. According to published GWAS data, *SNCA* is the most vital risk locus in PD patients. CRISPR/Cas9 technology was utilized in hESC cell lines to create *SNCA*^±^ and *SNCA*^−/−^ cell lines by *SNCA* gene KO, which is responsible for pathogenic α-synuclein aggregation. They were allowed to differentiate into midbrain dopaminergic neurons and treated with recombinant α-synuclein-preformed fibrils to initiate the formation of α-synuclein protein aggregates. Wild-type neurons were vulnerable to protein accumulation; in contrast, *SNCA*^±^ and *SNCA*^−/−^ cells exhibited resistance to forming this pathological hallmark [39, 40].

MSCs were also utilized to establish a novel, efficient therapeutic strategy. One of the principal causes of PD is microglial activation, and its inhibition by soluble receptors for advanced glycation end-products (*sRAGE*) through umbilical cord blood-derived mesenchymal stem cells (UCB-MSCs) can be a suitable therapeutic option through anti-inflammatory activity [122-124]. *sRAGE*-producing UCB-MSCs were created using CRISPR/Cas9 and transplanted into the corpus striatum of rotenone-induced PD animals. These cells offer the advantage of continuous production of the sRAGE proteins. The neuronal cell death in the corpus striatum and substantia nigra was significantly minimized, proving the neuroprotective action of CRISPR Cas9-edited *sRAGE*-MSCs in rotenone-induced PD [125]. In another study, hUMSC-derived extracellular vesicles were proposed as an attractive carrier strategy since they exhibited favorable effects in PD by targeting autophagy and lysosomal dysfunction. Using hUMSC-EVs as a carrier, *miR-106b*, a possible miRNA biomarker for PD diagnosis, was efficiently delivered into pathological neurons. The results confirmed that EVs-*miR-106b* increases neuronal autophagy and ameliorates neuronal damage in PD mice by inhibiting the *CDKN2B* gene [126].

## Future perspective and conclusion

PD has a sophisticated pathophysiology originating from multiple metabolic pathways that are disabled due to either accumulation of detrimental proteins or the degeneration of the substrates required to maintain neuronal homeostasis. Gene editing-based treatment directs us to focus on correcting the dysfunctional gene at a molecular level rather than just relieving the patient from the motor and nonmotor symptoms, as seen in conventional drug treatment. Since its discovery in 2013, CRISPR Cas9 genome editing has undergone tremendous progress over the years. It facilitates various activities, such as stimulating or repressing gene expression, KO, knockin, genome-wide screening, genomic imaging, designing disease models, and facilitating gene correction in one place. The supremacy of the CRISPR/Cas9 system over other gene editing techniques has attracted significant investments in the recent past. A study enumerated that the global CRISPR gene editing market was valued at $846 million in 2019 and is expected to reach $10,825 million by the end of 2030. CRISPR/Cas9 has excellent possibilities to explore in stem cell and regenerative research, like identifying the best gene editing targets and superior gene editing vehicles to understand the mechanism of complicated neurological disorders and the rationale for complex neuronal connections. CRISPR/Cas9 was used to successfully develop personalized cell therapy for sickle cell anemia and β-thalassemia by editing ES cells and patient-derived stem cells. Researchers are trying to utilize this 'super-tool' in developing therapies against HIV, retinoblastoma, Werner's syndrome, and neurodegenerative disorders like PD. A bright future with CRISPR/Cas9 revolutionizing research in combination with regenerative medicine by improving genomic and epigenomic editing in disease pathology is envisioned.

## Data Availability

Not applicable.

## Abbreviations

AAV: Adeno-associated Virus
AdV: Adenovirus
ALP: Autophagy lysosomal pathway
Cas9n: Cas9 nickase
CBP: Cripto-blocking peptide
CMA: Chaperone-mediated autophagy
CPP: Cell-penetrating peptide
CRISPR/Cas9: Clustered, regularly interspaced short palindromic repeats
DaN: Dopaminergic neuron
DJ-1: Daisuke-Junko-1
DMD: Duchenne muscular dystrophy
DNMT1: DNA methyltransferase 1
DSB: Double-stranded breaks
ES: Embryonic stem
ESC: Embryonic stem cell
GBA: Glucocerebrosidase gene
GCase: Glucocerebrosidase enzyme
GDNF: Glial cell line-derived neurotrophic factor
GHSR: Growth hormone secretagogue receptor
GMF: Glia maturation factor
GNP: Gold-based nanoparticle
hAMSC: Human primary adipose-derived mesenchymal stem cell
HDAC: Histone deacetylase
HDR: Homology-directed repair
hEDSC: Human-endometrial-derived stem cells
hESC: Human embryonic stem cell
HNH: Histidine-Aspargine-Histidine
hucMSC: Human umbilical cord mesenchymal stem cell
IGF1: Insulin-like growth factor 1
iPSC: Induced pluripotent stem cell
LiCl: Lithium chloride
LNP: Lipid-based nanoparticle
LRRK2: Leucine rich repeat kinase 2
LV: Lentivirus
MIDN: Midnolin
MPTP: 1-Methyl-4-phenyl-1,2,3,6-tetrahydropyridine
MSC: Mesenchymal stem cell
NSC: Neural stem cell
NUC: Nuclease domain
PAM: Protospacer adjacent motif
PD: Parkinson’s disease
PI: PAM-interacting domain
PINK1: PTEN-induced putative kinase
PK2: Prokineticin
PKCδ: Protein Kinase Cδ
PNP: Polymer-based nanoparticle
PRKN: Parkin RBR E3 Ubiquitin Protein Ligase
PSP: Persephin
pUb: PhosphoS65-ubiquitin
REC: Recognition site (gRNA binding)
ROS: Reactive oxygen species
SaCas9: Staphylococcus aureus Cas9
SEZ: Subependymal zone
SGZ: Subgranular zone
SNCA: α-synuclein
SNCT: Somatic cell nuclear transfer
SNP: Single nucleotide polymorphism
SNpc: Substantia nigra pars compacta
SpCas9: Streptococcus pyogenes Cas9
sRAGE: Soluble receptors for advanced glycation end-products
TALEN: Transcription activator-like effector nucleases
TH: Tyrosine hydroxylase
UPS: Ubiquitin proteasomal system
WED: Wedge domain
YOPD: Young-onset PD
ZFN: Zinc-finger nucleases
